# New Frontiers in Biosafety and Biosecurity

**DOI:** 10.3389/fbioe.2021.727386

**Published:** 2021-07-21

**Authors:** Alan Raybould

**Affiliations:** Global Academy of Agriculture and Food Security and the Innogen Institute, Old Surgeons’ Hall, University of Edinburgh, Edinburgh, United Kingdom

**Keywords:** sustainable development, regulation, innovation, biotechnology, decision-making

## Introduction

Biotechnology has great potential to contribute to sustainable development. Over the past 18 months, it has enabled rapid deployment of methods to detect, treat and protect people against infection by SARS-CoV-2 (Baek et al., 2020; Beigel et al., 2020; Voysey et al., 2021). In addition, gene editing is promising to revolutionize medicine, public health, agriculture and manufacturing through, among other things, the treatment of hereditary diseases, the control of agricultural pests and vectors of dangerous human pathogens, the breeding of crops for healthier diets and livestock for greater animal welfare, and the production of organisms for industrial biotechnology that produce raw materials that may replace fossil fuels in the manufacture of numerous products (Barrangou and Doudna, 2016; Collins, 2018; Ricroch, 2019; Clarke and Kitney, 2020).

Nevertheless, application of biotechnology could cause severe harm if the associated risks are not well managed. Gain-of-function research may increase our knowledge of pathogen evolution; however, it may also cause catastrophic effects if laboratory containment fails or if the new knowledge is used to develop biological weapons (Duprex et al., 2015). Treatment of disease using gene editing, particularly through heritable modifications, raises numerous questions about the bearing of inter-generational risks and the possible exacerbation of health inequalities (Vasiliou et al., 2016). And the use of biotechnology in agriculture remains controversial over 25 years after genetically modified (GM) crops were first grown commercially. Supporters point to reduced pesticide use, greater carbon sequestration and increased yield and profitability for farmers who grow GM crops (Brookes and Barfoot, 2018). By contrast, critics claim that the use of GM crops perpetuates harmful environmental and social consequences of industrial agriculture (Wilson et al., 2021).

To realize the potential of biotechnology, society must envisage biosafety and biosecurity as more than simply containment of organisms that have been bioengineered. Biosafety and biosecurity should seek to enable continuous improvement in policy- and decision-making to optimize the balance between opportunity and risk in using biotechnology to find sustainable solutions to societal problems. I discuss three new frontiers that must be opened to achieve this aim: political leadership in making and justifying choices about the use of biotechnology for sustainable development; regulations that encourage innovation; and responsible innovation by businesses and responsible engagement by civil society.

## Frontier 1: Policy Leadership

“Following the science” is a phrase commonly used by governments during their responses to the spread of SARS-CoV-2 (Stevens, 2020). It implies that “correct” decisions are reached solely by rigorous scientific analysis and reliable data. However, good decision-making “depends above all on sound ethical reasoning that ascribes value and normative judgement to empirical facts” (Cristina de Campos-Rudinsky and Undurraga, 2021). Data on the reliability of tests for a virus and the efficacy and safety of a vaccine alone cannot determine whether particular people ought be tested or vaccinated. Such decisions require ethical and political evaluation of what these procedures are intended to achieve in circumstances where choices must be made. Once a trade-off has been identified–for example, between cancer diagnoses and treatment for COVID-19 during the SARS-CoV-2 pandemic (Dinmohamed et al., 2020)—data on the performance of tests and vaccines can contribute to the design of options for achieving the best outcome. However, the definition of “best outcome” remains a political and ethical choice, not a scientific discovery.

“Following the science” is convenient for decision-makers who wish to avoid controversy over the reasoning behind the choices they have made. Prioritizing COVID-19 treatment over cancer diagnoses is one example. Another is decision-making about whether to permit cultivation of GM crops in the EU. Here decisions are regularly postponed to wait for new studies that ostensibly aim to reduce scientific uncertainty about the properties of a crop to a level where the correct decision becomes clear (Mastroeni et al., 2021). However, repeated failure to reach decisions seems to be more about the unwillingness or inability of decision-makers to formulate clear policy aims for GM crops; hence, they request more data as a delaying tactic rather than as an aid to decision-making (Devos et al., 2014; Mastroeni et al., 2021). Attempts to contract out decision-making to “the science” are bad for public policy as the values underlying choices are not debated, decisions appear arbitrary, and scientific advisors may be able to make policy decisions that are not theirs to make (Pielke, 2007; Raybould and Macdonald, 2018).

Opening the first new frontier for biosafety and biosecurity requires political leadership to stop hiding behind scientific advice and clearly define the trade-offs and justify the inevitable choices that must be made to maximize the sustainable development opportunities provided by biotechnology. There will be trade-offs between objectives; for example, reducing greenhouse gas emissions may be incompatible with increasing dietary choices. There will also be trade-offs in delivery of the objectives. Banning all biotechnology research may maximize short-term human safety but endanger it in the long term because medicine, agriculture and manufacturing are unable to innovate. Conversely, placing no restrictions on research may hasten the development of life-saving products but also increase the probability of existential damage to human civilization (Sears, 2020).

In such circumstances, political leadership must choose the balance between divergent objectives so that policy is co-ordinated and businesses know what kinds of product are required. Scientists should encourage political discussion of the role of biotechnology in enabling these choices and discourage attempts to avoid debate about choices through “following the science.” A corollary is that scientists should refrain from using scientific advice as “stealth advocacy” for their preferred policy choices (Pielke, 2007). Scientific advisors should provide options, including the use of biotechnology where suitable, for accomplishing agreed policy choices; they should not seek to close down debate by implying that certain policy choices are scientifically valid or invalid.

## Frontier 2: Regulation and Innovation

Active political leadership provides top-down setting of general objectives for biotechnology in sustainable development. By contrast, delivering these objectives requires bottom-up innovation in the application of biotechnology. Crucial to this task is whether the principal aim of biotechnology regulatory policy is elimination of risk or willingness to take acceptable risk based on the value of the opportunity. The former is sometimes described as the precautionary principle and the latter as the innovation principle (Bogner and Torgersen, 2018).

**FIGURE 1:**
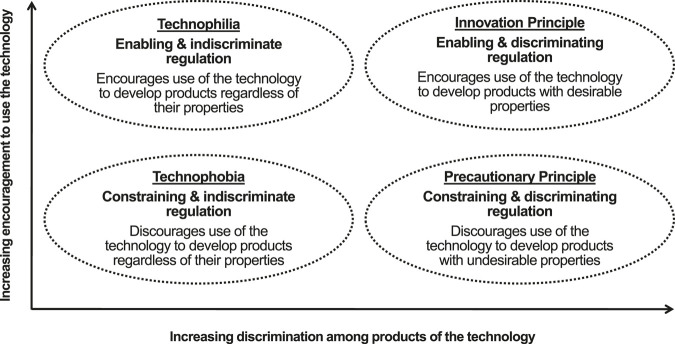
A conceptual classification of approaches to regulation developed from Chataway et al. (2006) and Raybould (2019).

Figure 1 shows different conceptual approaches to regulation of technology and how the innovation and precautionary principles differ. Regulation of medical (“red”) biotechnology seems to apply the innovation principle. While problems in implementation remain (Syrett, 2020), regulatory authorities for medicines recognize that regulations must encourage innovation as well as control risk (Nagai, 2019). To maintain a suitable balance between innovation and risk, regulation of medical biotechnology seeks timely adaptation to general trends, such as the increasing expectations of patients, rapid scientific developments, and changes in healthcare systems and the pharmaceutical industry (Eichler et al., 2015). In addition, decision-making is flexible, with authorities able to issue emergency use authorizations for products, such as SARS-CoV-2 vaccines and treatments, that provide countermeasures to public health crises (Eastman et al., 2020).

Regulation of applications of biotechnology to agriculture, public health and environmental protection (“green biotechnology”) seems to apply the precautionary principle or even technophobia. Evaluation of green biotechnology products focuses on detecting the potential to cause harm rather than deliver benefit. Indeed in EU regulatory evaluations, consideration of the potential benefits of GM crops is explicitly excluded (Bartsch, 2014). Decision-making is inflexible, with data requirements being slow adapt to advances in knowledge about the process of genetic modification (Herman et al., 2009) and familiarity with the types of product being evaluated (Raybould and Poppy, 2012; Bachman et al., 2021).

A precautionary approach to regulation of green biotechnology has stifled innovation. The product range is limited, comprising mainly herbicide-tolerant and insect-resistant GM commodity crops produced by a few large multinational companies (Bonny, 2017). Innovation is encouraged when a different regulatory approach is adopted. Argentina regulates gene-edited (GE) crops similarly to conventionally bred crops and the range of products and product developers is markedly greater than for GM crops (Whelan et al., 2020). Exempting GE crops from GM regulations is a good start in changing the focus of regulations for green biotechnology from precaution to innovation. Making the regulation of all green biotechnology more like that of red biotechnology would be even better and represents a second new frontier for biosafety and biosecurity.

## Frontier 3: Responsible Innovation and Engagement

A third new frontier for biosafety and biosecurity is enthusing civil society about the potential for biotechnology, particularly green biotechnology, to deliver sustainable development. If large sections of society are hostile to biotechnology, political leaders may be unwilling to make the case for its role in sustainable development, and regulatory systems are likely to become even more focused on precaution than innovation, thereby undermining progress on the other new frontiers.

Eliminating hunger is a vital sustainable development goal. However, GM crops as a solution to mass starvation may have been oversold, conveying a rather threatening and pessimistic tone; in effect, product developers have been saying allow us to use biotechnology or millions of people will starve (Raybould, 2019). Such messages of “doom and gloom” tend to create apathy, not inspiration (Knowlton, 2017). It is unsurprising, therefore, that people are sceptical or even cynical about the motives of GM product developers and the opportunities for green biotechnology to contribute to environmental, social and economic sustainability, even if they accept that it may make existing systems more productive in the short term.

Creating optimistic messages that green biotechnology can reduce hunger while also changing aspects of current production systems that people dislike is crucial. An interesting example is a paper by Kwon et al. (2020) who used gene editing to make tomato plants more compact and earlier yielding. Rather than presenting the crop as a potential improvement for existing tomato production, they discussed how it could be used in hydroponic vertical farms. Industrial (“white”) biotechnology may similarly contribute to changing the societal perception of biotechnology by developing products that replace meat from livestock (Rischer et al., 2020).

Certification and standards are useful for product developers wanting to go beyond regulatory compliance as a way to back up claims about sustainability. The recently launched British Standards Institution Responsible Innovation (RI) Guide provides a structured process for product developers to demonstrate that they have taken action to minimize the potential harmful effects and maximize the potential benefits of their products (Tait et al., 2021). One can envisage compliance with sustainability standards becoming a part of such RI exercises. However, current sustainability schemes, particularly in agriculture, tend to exclude products of biotechnology (Williams et al., 2018). The lost opportunities caused by prejudice against biotechnology in the “sustainability certification industry” emphasizes that everyone, not just product developers but also NGOs and other elements of civil society, has a duty to behave responsibly in debates about the use of biotechnology (Raybould, 2019).

## Conclusion

Achieving sustainable development will be extraordinarily difficult, hence all the different colours of biotechnology should be evaluated for potential to contribute to its realization. Regarding biosafety and biosecurity as being more than the minimization of risk from potentially dangerous organisms will be key to this enterprise. Biosafety and biosecurity should be reimagined as techniques for optimizing the balance between opportunity and risk in the application of biotechnology to sustainable development. Achieving this objective requires co-ordinated change on several fronts: political leadership to make and justify policy choices that maximize opportunities for biotechnology to find sustainable solutions to societal problems; regulations that consider the need to innovate as being at least as important as the need to be precautionary; and civil society that is prepared to engage responsibly in policy debates about the potential contribution of biotechnology to sustainable development. The final element may be the most difficult to achieve as there is considerable vested interest in defining sustainability as being fundamentally incompatible with the use of biotechnology.
